# Assessment of client satisfaction in labor and delivery services at a maternity referral hospital in Ethiopia

**DOI:** 10.11604/pamj.2014.17.76.3189

**Published:** 2014-01-31

**Authors:** Tadele Melese, Yirgu Gebrehiwot, Daniel Bisetegne, Dereje Habte

**Affiliations:** 1 University of Botswana-School of Medicine, Gaborone, Botswana; 2 Addis Ababa University, Department of Obstetrics & Gynecology, Ethiopia; 3 Consultant Obstetrician and Gynecologist, Addis Ababa, Ethiopia

**Keywords:** Client satisfaction, patient satisfaction, maternal care, Addis Ababa, Ethiopia

## Abstract

**Introduction:**

Patients perception about service quality shapes their confidence with regard to use of the available health care facility. This study is aimed to assess the client`s satisfaction in a maternal health care setting.

**Methods:**

This is an institution based cross sectional descriptive study. A total of 423 postpartum women were interviewed. Data was analyzed using SPSS version 20 statistical package.

**Results:**

The proportion of mothers who are completely satisfied with health care ranges between 2.4 to 21%. Pain control was the poorest source of satisfaction with 82% reporting dissatisfaction. Provider's communication with clients yielded complete satisfaction rates ranging between 0.7 to 26%. Inadequate information about the drug prescribed and explanation of procedures to be done to the client were found to be major causes of dissatisfaction. The complete satisfaction rate with environmental factor of the hospital was between 3.3 to 40.2%. Age of the client, educational status, income of the client and client's address away from Addis Ababa were found to be the predictors of client satisfaction. Provider's attitude and communication, as well as longer duration of stay in the ward were independent predictors of client satisfaction.

**Conclusion:**

Pain management, client privacy and client provider communication need to be addressed to ensure the satisfaction of maternity clients. The clients need to be involved in the management of their own health problems.

## Introduction

Patient satisfaction is defined as the individual's positive evaluation of distinct dimension of health care[1]. Patient satisfaction questionnaires have their origin in two separate developments to improve compliance and assess consumerism[2]. Patients with lower expectations tend to be more satisfied. Demographic characteristics such as age, educational attainment, and socio-economic status are some of the factors considered to influence measured satisfaction ratings[3, 4]. The reasons for measuring patient satisfaction include describing health care service from the patient's perspective, measurement of the process of care and evaluation of care as a function of patient satisfaction.

Satisfaction is a multidimensional construct involving interpersonal manner, quality of care, accessibility or convenience, finance of care, consistency, physical environment and availability[4–6]. It is not important whether the patient is right or wrong but what is important is how the patient felt[7]. Patient satisfaction is the result of congruent or incongruent orientation towards a certain health care in the community [8–13]. Patient with higher satisfaction are more likely to stick to medical recommendations[14]. Educated groups are less satisfied than less educated groups, because of higher expectations. Patients with higher social class are often more satisfied. Nations have differences in satisfaction levels based on their economic development and health care systems[15, 16]. Professional competence and quality of communication are the key predictors of consumer satisfaction [17–20]. More than 200 million women get pregnant annually, and about 15% develop complications that will require skilled obstetric care[21]. All women need good quality maternal health services to safeguard the lives of themselves and their and unborn children[22, 23]. High -quality maternal care should be a continuum that spans from the pre-pregnancy to the postpartum period and in which women and health providers are partners in the care provision[20, 23].

In Ethiopia, efforts are being made to make health institutions client oriented. However evidence is still inadequate on the patient`s perspective. This study is conducted with the aim of identifying the levels of client`s satisfaction and its determinants in maternity care at a referral hospital.

## Methods

### Study setting and sample

This is an institution based cross sectional study undertaken from November-December, 2006 at Gandhi Memorial Hospital (GMH). GMH is a university affiliated maternity referral hospital in Addis Ababa, Ethiopia. Clients are referred from the lower level hospitals and health centers for specialty care in the GMH. Clients who have no regular follow up at GMH and emergency patients are also treated if they present to the hospital without referral.

### Sample size and sampling procedure

Epi-Info 6.04d software was used in the calculation of sample size. The assumptions for sample size calculation were a 50% satisfaction (to attain the maximum sample size), 5% level of significance and 5% margin of error. To compensate for non-response, 15% of the calculated sample size was added. The sample size required was 442 participants while data was obtained from 451 respondents in the actual data collection. All respondents were interviewed on discharge in a separate room. Each consecutive case admitted in the wards was included in the study. Of 451 completed questionnaires, 423 have been analyzed as the rest (6.2%) were excluded because of incompleteness.

### Measurement

The questionnaire was composed of two parts: socio-demographic section and 20 satisfaction related variables which came up with a high internal consistency (cronbach`s alpha= 0.82). A five point Likert scale ( 1=completely dissatisfied ;2=dissatisfied; 3=neutral;4=satisfied and 5=completely satisfied) was used to rate satisfaction with various aspects of health care. The satisfaction variables were grouped into four categories: healthcare (five questions); health workers communication (six questions); attitude of the health worker (five questions) and the hospital physical environment (four questions). An exit interview was performed by trained nurses in casual dress with no affiliation to GMH.

### Data analysis

Data was entered and analyzed using SPSS version 20 statistical package. P-value of 0.05 was taken for statistical significance. Frequency and percentage were computed to describe the findings. Chi-square test and Odds Ratio with 95% Confidence Interval were computed in order to test for association. Multiple logistic regression analysis was made using the stepwise backward option for the four classes of satisfaction separately. The outcome variable was satisfaction to health service delivery while the explanatory variables were socio-demographic and health care related variables. Adjusted Odds Ratio (AOR) with 95% Confidence Interval (CI) was computed. Cronbach's Alpha was applied in order to evaluate the instrument. The satisfaction scores for each item are grouped in either satisfied or not satisfied to create a binary outcome variable.

### Ethical issues

Ethical approval was secured from the Department of Gynecology & Obstetrics Research Committee and School of Medicine Institutional Review Board. Informed consent was obtained from each study participant before the administration of questionnaires after explaining the purpose of the study. Personal identifier information such as name was not recorded in the questionnaire.

## Results

Table 1 shows the socio-demographic and maternity care related characteristics of 423 clients involved in this study. In this study, 10.6 % of the clients are teenagers while only 9.5% are single mothers. Three mothers (0.7%) are forming the group of widow or divorced. The remaining participants are married and supported. Four-fifth (81%) of the study population is Christians. With regard to education, 21% were not educated at all while 48% of the clients attained at the level of secondary school or above. The large majority of the clients (85.3%) are from Addis Ababa (the capital of Ethiopia). The pregnancy was planned and wanted by three quarter and two-third of the clients was primi-para. Two-thirds of the delivery was attended by doctors: 73.5% of the delivery being spontaneous vaginal delivery (SVD), 18.4% of the clients delivered by cesarean section (C/S) (both emergency and elective) and 94.8% of the clients having alive pregnancy outcome. Of the total clients, 26.2% of the cases were discharged from the hospital within 12 hours of admission. Over three quarters (77.3%) of the clients are paying while the rest are getting free service.

**Table 1:** Socio-demographic and maternity care related characteristics of study participants

Variable (n = 423)	Frequency	Percentage
**Age in years**		
15-19	45	10.6
20-24	148	35.0
25-29	138	32.6
30-34	69	16.3
35-39	23	5.4
**Marital status**		
Single	40	9.5
Married	380	89.8
Divorced	2	0.5
Widowed	1	0.2
**Religion**		
Orthodox Christian	307	72.6
Protestant Christian	36	8.5
Muslim	75	17.7
Others	5	1.2
**Education**		
Does not read & write	89	21
Primary	130	30.7
Secondary	151	35.7
Above secondary	53	12.5
**Income** ***(1USD=8.75ETB)***		
Below 600 ETB	244	57.7
600 ETB and more	179	42.3
**Address**		
Addis Ababa	361	85.3
Out of Addis Ababa	62	14.7
**Pregnancy status**		
Wanted	319	75.4
Unwanted	104	24.6
**Parity**		
Primi-para	252	59.6
Multi-para	181	40.4
**Delivery attended by**		
Physician	286	67.6
Nurse	45	10.6
Unknown	92	21.7
**Mode of delivery**		
SVD	311	73.5
Instrumental delivery	34	8.0
Elective C/S	19	4.5
Emergency C/S	59	13.9
**Gender of health care provider**		
Male	345	81.6
Female	78	18.4
**Hours of stay in the hospital**		
1-12 hours	111	26.2
13-24 hours	182	43
25-48 hours	42	9.9
Above 48 hours	88	20.8
**Treatment fee**		
Paying	327	77.3
Free	96	22.7
**Pregnancy outcome**		
Alive	401	94.8
Dead	22	5.2

### Client satisfaction to maternity care

Table 2 presents the proportion of respondents satisfied with each of the 20 items as well as their mean satisfaction score. The client's satisfaction was measured using a satisfaction scale of completely dissatisfied 1 to completely satisfied 5. Of the 20 items, a proportion of complete satisfaction above 25% was recorded for waiting time to see provider (35.5%), provider listens to your worries (26%), respect by provider (41.6%), respect by nurse (40.7%) and amount of freedom in the ward (40.2%). Lower proportion of complete satisfaction was reported for pain control (2.4%), providers’ explanation about client's condition (4.5%), provider's explanation about medications (0.7%), information given about the procedures (0.9%), quality of meal (3.3%) and facility cleanliness (3.8%). The mean satisfaction score for health care related items was at a higher scale (3.90-4.14) except for pain control (2.33). The mean satisfaction score for health worker attitude variables ranged from 3.85 to 4.24. Health workers communication and environment categories recorded a relatively lower mean satisfaction score: the lowest score in the two groups being provider's explanation about medications (2.44) and facility cleanliness (3.43) respectively.

**Table 2:** Mothers level of satisfaction towards perinatal care (n=423)

Variables	Completely satisfied (%)	Satisfied (%)	Mean satisfaction score*
**Health care**			
Time spent with provider	21.0	71.9	4.08
Medical care received today	8.3	80.1	3.90
Care received from Nurse	11.8	81.6	4.02
Waiting time to see provider	35.5	52.7	4.14
Pain control	2.4	15.6	2.33
**Health workers communication**			
Provider explained your condition	4.5	62.4	3.45
Provider explained about drug	0.7	24.6	2.44
Provider listens to your worries	26.0	62.6	4.06
Nurse listens to your worries	20.3	69.7	4.04
Information about procedure & exam	0.9	45.4	3.02
Information at discharge	10.4	50.1	3.32
**Health workers attitude**			
Courtesy, respect by Doctor	41.6	48.2	4.23
Courtesy, respect by nurse	40.7	50.1	4.24
The way staff treated you	10.2	75.7	3.86
The way staff treated family or companion	10.9	74.5	3.85
Acceptance of opinion by staff	6.9	83.5	3.90
**Environment**			
Amount of freedom in the ward	40.2	57.9	4.37
Amount of privacy in the ward	9.2	69.7	3.70
Quality of meal	3.3	59.6	3.49
Facility cleanliness	3.8	64.1	3.43

* The satisfaction score was computed with scores as follows: Completely satisfied = 5; Satisfied = 4; Neutral = 3; Dissatisfied = 2; completely dissatisfied = 1.

### Multivariate Analysis

Binary logistic regression analysis was made for four categories of independent variables (Table 3). Mothers with secondary level education and hospital stay more than 48 hours were more likely to be satisfied with the health care (AOR 2.08, p<0.05 and 4.32, p<0.001) where as those delivered by a nurse were less likely to be satisfied with the health care (AOR 0.27, p<0.05). Satisfaction to health workers’ communication was also more likely among secondary education group (AOR 3.20, p<0.01) and less likely among mothers with unwanted pregnancies (AOR 0.46, p<0.05). Mothers in the age group 25-29 and 30-34 years were more likely to have satisfaction with providers attitude as compared to the 15-19 years groups (AOR 2.69, p<0.05 and 2.68, p<0.05). Participants above secondary level of education were less likely to have satisfaction with providers attitude (AOR 0.30, p<0.05). Older age groups, better income, unwanted pregnancy status, instrumental delivery and emergency caesarian section were negative predictors of satisfaction with the hospital environment. Mothers residing outside Addis Ababa were more likely to be satisfied with the environment (AOR 2.00, p<0.05).

**Table 3:** Factors significantly associated with mothers’ satisfaction: binary logistic regression analysis (n = 423)

Variable	Health care	Communication	Attitude	Environment
	OR (95% CI)	OR (95% CI)	OR (95% CI)	OR (95% CI)
**Age in years**				
15-19			1.00	1.00
20-24			1.18(0.54,2.58)	0.44(0.20,0.96)*
25-29			2.69(1.20,6.01)*	0.40(0.18,0.89)*
30-34			2.68(1.08,6.65)*	0.39(0.16,0.93)*
35-39			2.12(0.65,6.90)	0.20(0.06,0.65)**
**Education**				
Does not read & write	1.00	1.00	1.00	
Primary	1.21(0.60,2.41)	1.86(0.86,4.00)	0.85(0.46,1.57)	
Secondary	2.08(1.08,4.03)*	3.20(1.55,6.59)**	0.81(0.43,1.53)	
Above secondary	1.32(0.56,3.11)	2.09(0.85,5.13)	0.30(0.12,0.74)**	
**Income**				
Below 600 ETB			1.00	1.00
600 ETB and more			2.07(1.28,3.36)**	0.44(0.28,0.67)***
**Address**				
Addis Ababa				1.00
Out of Addis Ababa				2.00(1.07,3.71)*
**Pregnancy status**				
Wanted		1.00		1.00
Unwanted		0.46(0.25,0.87)*		0.53(0.32,0.88)*
**Parity**				
Primi-para	1.00		1.00	
Multi-para	1.53(0.96,2.45)		0.66(0.41,1.07)	
**Delivery attended by**				
Physician	1.00			
Nurse	0.27(0.10,0.74)*			
Unknown	0.54(0.29,1.01)			
**Mode of delivery**				
SVD				1.00
Instrumental delivery				0.12(0.04,0.34)***
Elective C/S				0.81(0.30,2.14)
Emergency C/S				0.46(0.25,0.84)*
**Hours of stay in the hospital**				
1-12 hours	1.00			
13-24 hours	1.26(0.67,2.35)			
25-48 hours	2.13(0.92,4.92)			
Above 48 hours	4.32(2.21,8.43)***			
**Treatment fee**				
Paying				
Free				
**Pregnancy outcome**				
Alive				1.00
Dead				2.40(0.85,6.78)

* P < 0.05

** P < 0.01

*** P < 0.001

## Discussion

The study has shown important findings in relation to client satisfaction in different aspects: health service, health workers communication and attitude, and hospital environment. Client`s poor pain control was found to be a leading cause of client dissatisfaction in this study. This study has shown an overall satisfaction to waiting time of 88.2% (of which 35.5% had complete satisfaction) which is slightly higher than the previous study in rural Ethiopia which was 75.5 %( Eskindire et al, 2004)[24] and much higher than another study from Slovenia that reported 26% as excellent (Janko Kersnk, 2000)[25]. As this study was conducted in a maternity hospital where laboring mothers constitute the majority of cases of such unit, a relatively higher satisfaction might be because of the nature of obstetric emergency care that women will have on arrival. Because of such emergencies the waiting time will be reasonably shorter. Even the 12% of mothers who are not satisfied might be those mothers with increased obstetric complication which is not explored in our study.

Consultation time, medical care by the attending doctor and nursing care are rated to have higher rate of overall satisfaction of 92.9%, 88.4% and 93.4% respectively. However complete satisfaction for consultation time, medical care by the attending doctor and nursing care were in the lower range: 21%. 8.3% and 11.8% respectively. Our finding seems to be better than the previous findings of outpatient care in Ethiopia with 80.6% satisfaction with consultation time[24]. Another study in the northern Ethiopia also revealed 61.9% satisfaction[26]. It is also higher than the finding in Slovenia that reported 51.6% satisfaction with waiting time [25]. Since this study was done on an inpatient service primarily dealing with different obstetric emergencies, striving towards complete satisfaction should be the goal of health institutions of developing countries like ours.

The clients seem to have higher satisfaction with regards to the doctor and the nurse communication with overall satisfaction rate of 88.6% and 91%, but the complete satisfaction rate contributing only 26% and 20.3% respectively. This result needs careful interpretation due to the fact that a women responding to be just satisfied might have given a positive response for merely having an alive and healthy baby. Explanation of the patients’ problem to the patient, information and counseling to the patient on discharge, explanation about examination or procedure to be done, and information to the patient on drugs prescribed to the patient has yielded a general satisfaction rate with decreasing order; 66.9%, 60.4% , 46.3% and 25.3%. However the complete satisfaction rates of such crucial communication variables were a maximum of 10.4% and the minimum benign 0.7%. There are evidences that provider communication to the client have a significant impact on client satisfaction and the finding in our study would alert the health care system to design a client friendly approach to enhance communication[27, 28]. Studies have demonstrated that client satisfaction is largely influenced by the involvement of the client on the decision of her treatment and the quality of client-care provider relationship. In our study it was found to be an area of major concern on such dimensions, especially the clients involvement in the decision making process in every aspect of their care[29].

Attitude of the health workers was found to have a relatively higher satisfaction score. Courtesy by the doctor and the nurse has yielded a complete satisfaction rate of 41.6% and 40.7% while half of the clients reported to be just satisfied. This finding is higher than the findings in Sri Lanka which was 31.4% and 21.1 %( Upul Senarath 2006). But the way the companion of client were treated by the staff and acceptance of clients’ opinion by the care provider yielded complete satisfaction rate of 10.9% and 6.9% respectively. Such lower satisfaction with respect to the companion's treatment and opinion acceptance might ultimately affect client compliance and treatment outcome. Hanna-Leena Melender revealed that pregnant mothers have higher expectations of the characteristics and attitude of care providers in order to have satisfaction in their labor and delivery process. Clients need a clear understanding of any obstetric interventions and clear consent for the intended procedures. Clients also need a proper interpretation of their opinion in the process of their care[27, 28].

Environmental factor was found to be one of the major factors affecting client satisfaction outcomes. Of these dimensions, the clients’ freedom in the ward yielded a complete satisfaction of 40.2%. Client privacy has complete satisfaction rate of 9.2% which is just comparable to the 10.8% by level in Sri Lanka. Respect for privacy is found to be a major predictor of satisfaction from previous studies in northern Ethiopia and Bangladesh (Jorge Mendoza Aldana, 2001)[26, 30]. Quality of meal and sanitation was found to have complete satisfaction rate of 3.3% and 3.8% respectively. This finding is lower than the finding in Sri Lanka which reported 10.8% satisfaction level with cleanliness. To address such compromised patient care, revisiting the patient care policies and strategies in the health care delivery system is important[27].

Clients whose educational level is secondary school were more likely to be satisfied with attitude of the provider (OR=2.08) and providers communication (OR= 3.2) in conformity with the finding by Ibrahim (2008),[31]. However other studies reported less satisfaction of patient with increasing educational background whereas higher satisfaction was seen among the lower education groups which was ascribed to their less expectation. Generally the finding in our study can be explained by a better understanding between the secondary school level clients and the care providers[32]. Mothers who came out of the capital (Addis Ababa) were more satisfied with the physical environment. This finding is similar to the findings in Brazil, where patients from rural Brazil had shown higher satisfaction with the health care delivery as compared to urban residents. This may be due to less expectation by the rural clients because of their previous experience locally where the health facilities might not be of good standard as of the urban set up[33]. From this study higher income clients demonstrated higher level of satisfaction with health care provider's attitude and lower level of satisfaction with the hospital environment. A study conducted in a similar setting revealed that low income groups had higher satisfaction in relation to physical environment of the hospital[27]. There is also a finding from other studies which reported higher satisfaction rates among the higher socioeconomic class or those who can pay more than others. But the previous studies tried to reflect that patient from higher income class might be able utilize the existing service maximally[26, 34].

Clients who stayed longer in the hospital tended to have higher satisfaction with the health care. It is not in conformity with a study finding where longer stay in the hospital was associated with lower satisfaction rate especially with patient comfort, cleanliness of the physical environment and patient visiting[35]. The finding of our study may be a result of mother's perception of getting a better care through longer stay and clearing the fear of other complications as a result of early discharge from the hospital. A study done in Australia revealed that mothers feel less confident about the care of their babies and they feel uncomfortable about the possibility of having new medical complication after early discharge. They assume that overnight stay in the hospital after delivery is inadequate. Our finding might be explained in a similar fashion in the background of nonexistence of home based nursing or midwifery care in our setup [36]

## Conclusion

Medical care is generally satisfactory, but facility cleanliness and staff discipline are found to have its own impact on the clients concern about quality of care they are getting. In general they are also interested on the physical set up and meal quality. The inadequate pain control remains to be a major problem and is a main cause of patient dissatisfaction. Client-provider communication was found to be sub-optimal. Loss of privacy, poor meal quality and facility cleanness had negative impact on satisfaction level. We recommend that these are the areas to be revised in order to make our clients be satisfied from the service delivery.
